# Evaluation of six months sputum culture conversion as a surrogate endpoint in a multidrug resistant-tuberculosis trial

**DOI:** 10.1371/journal.pone.0200539

**Published:** 2018-07-19

**Authors:** Paul Meyvisch, Chrispin Kambili, Koen Andries, Nacer Lounis, Myriam Theeuwes, Brian Dannemann, An Vandebosch, Wim Van der Elst, Geert Molenberghs, Ariel Alonso

**Affiliations:** 1 Janssen Pharmaceutica, Beerse, Belgium; 2 I-BioStat, Universiteit Hasselt, Diepenbeek, Belgium; 3 Johnson & Johnson Global Services, Raritan, NJ, United States of America; 4 Janssen Research & Development, Titusville, NJ, United States of America; 5 I-BioStat, KU Leuven, Leuven, Belgium; Instituto de Diagnostico y Referencia Epidemiologicos, MEXICO

## Abstract

The emergence of multidrug resistant-tuberculosis (MDR-TB), defined as *Mycobacterium tuberculosis* strains with *in vitro* resistance to at least isoniazid and rifampicin, has necessitated evaluation and validation of appropriate surrogate endpoints for treatment response in drug trials for MDR-TB. The trial that has demonstrated efficacy of bedaquiline, a diarylquinoline that inhibits mycobacterial ATP synthase, possesses the requisite features to conduct this evaluation. Approval of bedaquiline for use in MDR-TB was based primarily on the results of the controlled C208 Stage II study (ClinicalTrials.gov number, NCT00449644) including 160 patients randomized 1:1 to receive bedaquiline or placebo for 24 weeks when added to an 18–24-month preferred five-drug background regimen. Since randomization in C208 Stage II was preserved until study end, the trial results allow for the investigation of the complex relationship between sustained durable outcome with either Week 8 or Week 24 culture conversion as putative surrogate endpoints. The relationship between Week 120 outcome with Week 8 or Week 24 culture conversion was investigated using a descriptive analysis and with a recently developed statistical methodology for surrogate endpoint evaluation using methods of causal inference.

The results demonstrate that sputum culture conversion at 24 weeks is more reliable than sputum culture conversion at 8 weeks when assessing the outcome of adding one new drug to a MDR-TB regimen.

## Introduction

The use of surrogate endpoints in drug development as a basis for reaching conclusions about the benefits of therapy has been received with enthusiasm and concern [1–4]. Surrogates can hasten treatment benefits for patients when the surrogate proves to predict clinical benefit, but use of surrogates could result in the adoption of questionable therapies if insufficient rigor is applied in surrogate evaluation. This becomes paramount when assessing the effectiveness of adding an experimental drug to a regimen for treatment of multidrug resistant-tuberculosis (MDR-TB), defined as *Mycobacterium tuberculosis* resistant to at least isoniazid and rifampicin. Because tuberculosis drug trials are usually very long, valid surrogate endpoints measured during or at the end of treatment could reduce both the time and cost of assessing the efficacy of new regimens or drugs.

There has been much debate about biomarkers and surrogate endpoints in drug trials for drug-sensitive-tuberculosis (DS-TB) and MDR-TB. Treatment for DS-TB began as an 18-month regimen until the British Medical Research Council (BMRC) randomized controlled studies showed that a 6-month, short-course regimen was feasible [5,6]. Sputum culture conversion on solid media after 2–3 months of anti-DS-TB treatment has been proposed as a surrogate marker of relapse-free cure [7]. While this is likely the best dichotomous biomarker available, its statistical validity as a surrogate is questionable based on a re-analysis of the BMRC trial data [8,9]. Also, Phillips et al [10], in an analysis of the tREMoxTB trial, found that sputum-based markers poorly discriminate between favorable and unfavorable outcomes. In contrast, Wallis et al [11–13] have claimed that 2-month sputum culture status is a good predictor for outcome in DS-TB when combined with duration of treatment as an independent variable.

Design features of MDR-TB clinical trials are not always suitable for surrogate endpoint evaluation. Randomization needs to be preserved until the study end and treatment duration for each treatment group should preferably be kept the same in order to estimate the treatment difference on both the putative surrogate and long-term outcome. While some authors have chosen 8 weeks for surrogate assessment [14,15], there is growing evidence that for MDR-TB, sputum culture conversion at 24 weeks or later is of greater prognostic value for clinical outcome [16,17]. A key distinction to make is to differentiate between a prognostic marker and a surrogate endpoint. A prognostic marker relates to a clinical endpoint and is an indicator of treatment response. In contrast, a surrogate endpoint is intended to substitute for a clinical endpoint, predict clinical outcome, and is statistically evaluated [18]. So, while the association of week 24 culture conversion with clinical outcome may be stronger than earlier time points, this by itself does not necessarily demonstrate the statistical validity of the surrogate.

In a re-analysis of the 120-week bedaquiline (BDQ) Phase II trial (C208 Stage II) (ClinicalTrials.gov number, NCT00449644) [19], we examined the complex association between outcome and either Week 8 or 24 culture conversion as putative surrogate endpoints using a recent information-theoretic approach for statistical evaluation of surrogate endpoints that is based on causal inference [20].

## Methods

### C208 study design

The clinical trial demonstrating efficacy of BDQ in newly diagnosed patients with pulmonary MDR-TB was a randomized, placebo-controlled trial (C208) for which treatment assignment was not changed until study end [19,21]. Each site obtained approval of the study protocol from at least one (or more, if required by local regulations) independent ethics committee or institutional review board (S1 Table). The trial was conducted in accordance with the principles of Good Clinical Practice and the Declaration of Helsinki. All patients provided written informed consent before trial entry.

The trial consisted of two stages. The independent first stage was a single-country, placebo-controlled, randomized trial in a small group of MDR-TB patients (N = 47) to compare the safety and efficacy of adding BDQ for 8 weeks to a preferred five-drug MDR-TB regimen [21]. The second stage (main trial) was a multi-country, placebo-controlled, randomized trial in a larger group of MDR-TB patients (N = 160). C208 Stage II compared the efficacy and safety of BDQ given for 24 weeks (BDQ 400 mg once daily for 2 weeks, followed by 200 mg three times a week for 22 weeks) versus placebo when added to a preferred five-drug MDR-TB regimen that was given for 18–24 months [19]. While national treatment-program regimens were respected, the preferred five-drug background regimen was ethionamide, pyrazinamide, ofloxacin, kanamycin, and cycloserine [19].

In both stages, the primary endpoint was time to confirmed sputum culture conversion from positive to negative in liquid broth. Changes in the background regimen were allowed according to the results of drug susceptibility testing, because of unacceptable adverse events or supply interruption of the drugs. The background regimen was continued for 12–18 months after the planned end of BDQ treatment, with an anticipated ≥6-month treatment-free follow-up period. This allowed clinical outcome to be assessed at 104 weeks (26 months) in Stage I and 120 weeks (30 months) in Stage II after randomization.

**Fig 1:**
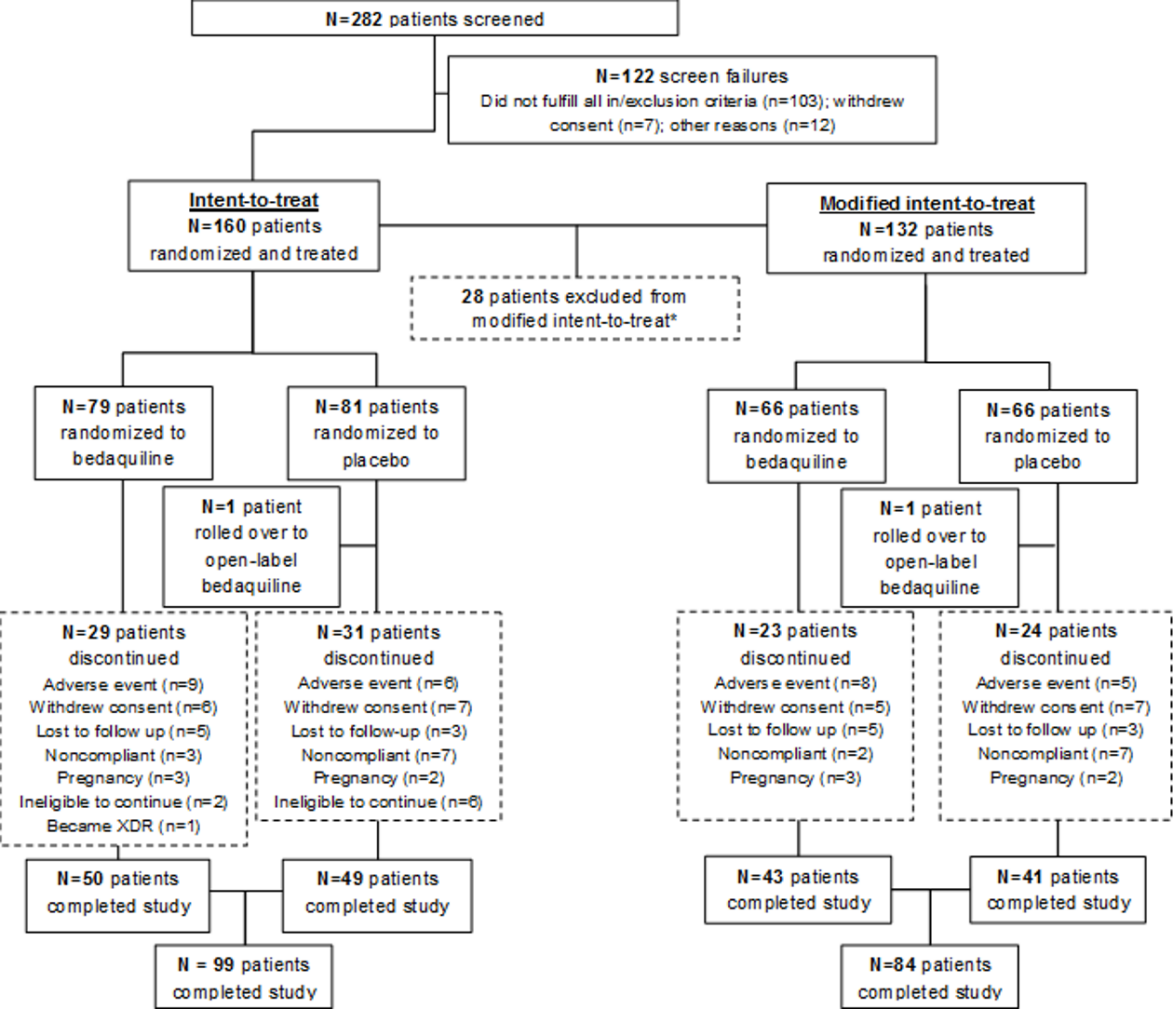
CONSORT flow diagram for the C208 Stage II trial [19]. *The modified intent-to-treat population was a subset of the intent-to-treat population that excluded nine patients (6 BDQ and 3 placebo) with Mycobacteria Growth Indicator Tube results that did not allow for primary efficacy evaluation (no evidence of culture positivity prior to first intake of blinded study drug or no results during the first 8 weeks after first intake), seven patients (3 BDQ and 4 placebo) infected with extensively drug-resistant tuberculosis, eight (4 BDQ and 4 placebo) with drug-sensitive tuberculosis, and four patients (0 BDQ and 4 placebo) for whom the multidrug-resistant tuberculosis status could not be confirmed.

While regulatory approval of the drug was obtained on the basis of ‘time to confirmed sputum culture conversion’ during the first 24 weeks in stage II, a binary endpoint of sputum culture conversion (achieved or not) was also used to demonstrate superiority of the BDQ-containing treatment group. Sputum culture conversion was evaluated after 24 weeks (treatment completion of BDQ or placebo) and again 24 weeks after the anticipated completion of the entire treatment regimen at the 120 week endpoint. The CONSORT flow diagram for the C208 Stage II trial is shown in Fig 1.

### Data collection and definition of endpoints in C208 Stage II

Spot sputum samples to assess the presence or absence of *Mycobacterium tuberculosis* were collected in triplicate at every visit and qualitative assessment was done in liquid medium (Mycobacteria Growth Indicator Tube [MGIT], Becton Dickinson) [19,22]. Visits were scheduled weekly during the first 8 weeks and biweekly until Week 24. From Week 24 onwards, visits were scheduled monthly until Week 36 and three-monthly thereafter until the end of the trial (Week 120).

#### Sputum processing

Sputum samples were decontaminated with N-acetyl-L-cysteine and sodium hydroxide solution and inoculated into MGIT tubes with the addition of oleic acid, albumin, dextrose and catalase (OADC) and polymyxin B, amphotericin B, nalidixic acid, trimethoprim and azlocillin (PANTA). MGIT tubes were incubated in the MGIT machine for 42 days, but were removed earlier if cultures flagged instrument-positive. The positive MGIT cultures were checked for growth of contaminating organisms by sub-inoculating the MGIT culture on a blood agar plate with overnight incubation. In addition, Ziehl-Neelsen staining was performed on each positive MGIT culture to check for the presence of acid-fast bacilli (AFB). Identification of the *Mycobacterium tuberculosis* complex was done on every positive MGIT culture using either the MPT64 antigen test or molecular tests. Quality control checking was done by growing the pan-sensitive H37Rv *Mycobacterium tuberculosis* strain on every new batch of MGIT tubes.

#### Aggregate scoring

Triplicate culture results were summarized prior to analysis into one single measure with values ‘negative’, ‘positive’, ‘contaminated’ or ‘missing’ [22]. This single aggregate measure was ‘negative’ when at least one of the three samples was negative and none were positive and was ‘positive’ when at least one of the three samples was positive.

#### Drug-susceptibility testing

Per protocol, drug-susceptibility testing (DST) was performed at a central laboratory (Institute of Tropical Medicine, Antwerp, Belgium) as previously described [19]. For DST, a culture was grown on Löwenstein–Jensen medium to generate enough colonies. DST for isoniazid, rifampicin, ethambutol, ofloxacin, ethionamide, kanamycin and capreomycin were performed on 7H11 agar (proportion method) and for pyrazinamide in the MGIT960 system. The quality control was performed by testing the pan-sensitive H37Rv *Mycobacterium tuberculosis* strain grown on 7H11 agar or using the MGIT960 system (pyrazinamide) with the same drug concentrations, each time the sensitivity of a clinical isolate was tested.

#### Study population and primary efficacy analysis

All patients contributing to the efficacy analyses were culture positive at baseline. Patients whose pre-randomization sputum sample was culture negative were excluded from the efficacy analysis, as were those whose culture was shown to be drug-sensitive or extensively drug-resistant, defined as MDR-TB with additional resistance to injectable second-line drugs (amikacin, kanamycin, or capreomycin) and fluoroquinolones. After exclusion of these categories of patients, the number of patients retained for primary efficacy analysis was 132 (66 patients in each treatment group) [19].

Regarding baseline susceptibility to drugs in the background regimen, 51 patients in the BDQ group vs 54 patients in the placebo group were infected with MDR-TB, and 15 vs 12, respectively, were infected with pre-extensively drug-resistant TB, defined as MDR-TB isolates also with resistance to second-line injectables or fluoroquinolones [19].

Sputum culture conversion at 8 weeks, 24 weeks, and at the end of the trial used the same criterion for confirmed conversion, i.e., the patient had to have at least two consecutive negative cultures at least 25 days apart (with no positive intermediate cultures). Patients who prematurely dropped out of the trial were considered failures from time of drop-out onwards, irrespective of whether they culture converted at the time they dropped out. We emphasize that this approach is consistent with the primary analysis as reported previously [19]. Since in this definition, data for patients who dropped out were imputed with the same outcome irrespective of their actual conversion at that time, using this missing = failure analysis was expected to increase the level of association (surrogacy) between the endpoints at different time points. Therefore, a sensitivity analysis using multiple imputation statistical techniques [23] to impute the data after drop-out was also conducted.

### Statistical evaluation of surrogate endpoints when both the surrogate and true endpoint are binary outcomes

In a single-trial setting, when both endpoints are expected to be normally distributed, a commonly used measure to evaluate surrogacy at the level of the individual patient is the adjusted association (γ), which is defined as γ = corr(S,T|Z), where corr is the Pearson correlation coefficient, S is the surrogate endpoint, T is the true endpoint, and Z is an indicator variable for treatment [2]. However, when we move away from settings in which both endpoints are normally distributed it is no longer clear how γ should be quantified. Importantly, it has now been clearly established that an association between the putative surrogate and the true endpoint does not guarantee the validity of the former. Indeed, the main idea behind the use of a surrogate endpoint is the prediction of the treatment effect on the true endpoint based on the treatment effect on the surrogate endpoint. Although desirable, a strong association between S and T is not enough to achieve this goal.

#### Individual causal association

Recently, a metric of surrogacy was proposed [20], the individual causal association (ICA), which uses information-theoretic concepts and a causal inference model for a binary surrogate endpoint and a true endpoint. The fundamental quantities that are used to determine the ICA are the Individual Causal Treatment Effects. The term ‘causal treatment effect’ refers to the causal effect of a given treatment on an outcome of interest like S and T. In the Neyman-Rubin ‘Potential Outcomes Framework’ of causality, an individual causal treatment effect is defined for each individual patient in terms of two ‘potential outcomes’. Each patient has one outcome that would manifest if the patient were exposed to the treatment and another outcome that would manifest if they were exposed to the control. The individual causal treatment effect is the difference between these two potential outcomes for the true endpoint and the surrogate endpoint, respectively (ΔT or ΔS). However, these individual-level causal treatment effects are unobservable because individual patients can only receive the treatment or the control, but not both. For any individual patient, ΔT and ΔS will be -1 (Harm), 0 (Equal), and 1 (Benefit), depending on how BDQ performs versus placebo on either endpoint.

The ICA is defined as the association between both individual causal treatment effects for the true endpoint and the surrogate endpoint, i.e., the association between ΔT and ΔS. As specified [20], the ICA always lies in the unit interval and has a simple and appealing interpretation in terms of uncertainty reduction. It takes a value of 1 if there is a deterministic relationship between ΔS and ΔT, and therefore ΔS predicts ΔT without error, i.e., knowing the treatment effect on the surrogate endpoint provides full information about the treatment effect on the true endpoint. In addition, when the ICA equals zero, both treatment effects are independent, and knowing the treatment effect on the surrogate endpoint does not inform about the treatment effect on the true endpoint. Like the individual causal treatment effects, ΔT and ΔS, the ICA cannot be estimated from the data directly. A two-step Monte-Carlo procedure was introduced to assess the value of the ICA [20]. The immediate consequence of ΔS and ΔT being non-identifiable is that estimation of the ICA results in a density rather than a fixed value.

While the interpretation of the ICA is straightforward, there is currently little guidance on how large it should be for a surrogate to qualify as acceptable. However, it is plausible to assume that when comparing two or more surrogates, the one with the largest ICA can be considered the best.

#### Surrogate predictive function

Another technique that has been developed to assist in interpreting the relationship between ΔT and ΔS is the Surrogate Predictive Function (SPF) [24]. As previously stated, S is a good surrogate for T, when ΔS can predict ΔT with a certain level of precision. Even though the ICA offers a general assessment of the surrogate predictive power, further insight can be gained from studying the conditional distribution of ΔT given ΔS. The general idea is to quantify the individual probabilities of all possible outcomes for the treatment effect on the true endpoint, given the treatment effect on the surrogate endpoint P(ΔT = i|ΔS = j) for i,j ϵ {-1,0,1}. All analyses are consistent and complimentary to the derivation of the ICA in the sense that it is based on the same two-step Monte-Carlo procedure as referenced above [20].

## Results

### Descriptive analysis

Denoting Week 8 culture conversion as the surrogate endpoint S_8_, Week 24 culture conversion as the surrogate endpoint S_24_ and favorable outcome at Week 120 as the true endpoint T, Tables 1 and 2 present the relationship between S_8_ and T and between S_24_ and T, respectively, for BDQ and placebo.

**Table 1:** Relationship between S_8_ and T for BDQ and placebo.

**BDQ**^**a**^		**Surrogate endpoint (S**_**8**_**)**	
		**No culture conversion**	**Culture conversion**
**True endpoint (T)**	**No culture conversion**	14	11
	**Culture conversion**	15	26
**Placebo**^**b**^		**Surrogate endpoint (S**_**8**_**)**	
		**No culture conversion**	**Culture conversion**
**True endpoint (T)**	**No culture conversion**	28	9
	**Culture conversion**	17	12

^a^ Odds ratio (OR) = 0.453; 95% CI: 0.165–1.249; p = 0.143

^b^ OR = 0.455; 95% CI: 0.159–1.306; p = 0.126

**Table 2:** Relationship between S_24_ and T for BDQ and placebo.

**BDQ**^**a**^		**Surrogate endpoint (S**_**24**_**)**	
		**No culture conversion**	**Culture conversion**
**True endpoint (T)**	**No culture conversion**	14	11
	**Culture conversion**	0	41
**Placebo**^**b**^		**Surrogate endpoint (S**_**24**_**)**	
		**No culture conversion**	**Culture conversion**
**True endpoint (T)**	**No culture conversion**	24	13
	**Culture conversion**	4	25

^a^ OR = 0.01; 95% CI: 0–0.190; p = 0.023;

^b^ OR = 0.087; 95% CI: [0.025–0.303]; p = 0.0001

A first observation is that there is a clear treatment effect on S_8_ (p = 0.005, 95% CI: 7.8–40.7%)_,_ S_24_ (p = 0.009, 95% CI: 5.7–36.7%) and on T (p = 0.036, 95% CI: 1.4–34.9%) using the Pearson χ^2^ test.

Secondly, the off-diagonal elements for the relationship between S_24_ and T shown in Table 2 (0 and 11) and (4 and 13), are generally smaller than for the relationship between S_8_ and T shown in Table 1 (15 and 11) and (17 and 9), indicating that S_24_ is in stronger ‘agreement’ with T compared with S_8_. This is also apparent from the odds ratio (OR) estimates and corresponding 95% confidence intervals, which are only significant for S_24_ in either treatment group. Do note that the OR for BDQ in Table 2 is determined using the Firth type estimate [25].

Looking specifically at the association between S_24_ and T, 11 patients in the BDQ group and 13 patients in the placebo group who were Week 24 responders were considered non-responders at endpoint. Of the 11 patients in the BDQ group, two died, five discontinued from the trial, and four reverted to positive culture. Of the 13 patients in the placebo group, five patients discontinued and eight patients reverted to positive culture. Additionally, four patients in the placebo group who were considered non-responder at Week 24 subsequently culture converted and were considered responders at Week 120. In contrast, all patients in the BDQ group who responded at Week 120 were already responders at 24 weeks. The analysis of the relationship between S_24_ and T shows a significant association for both BDQ and placebo patients; however, the association was stronger in the BDQ group than in the placebo group. This indicates that the addition of BDQ has a considerable impact as a greater proportion of patients culture convert and fewer relapse in the BDQ group. This change in relationship between surrogate and true endpoint upon addition of a new drug requires sound methodology to evaluate the statistical validity of the surrogates S_8_ and S_24_.

S2 Table presents the relationship between S_24_ and T for BDQ and between S_24_ and T for placebo, based on AFB rather than MGIT. In the AFB smear test, success was defined similarly compared to MGIT-based outcomes, i.e., the patient had to have at least two consecutive negative smears at least 25 days apart (with no positive intermediate smears). Patients who prematurely dropped out of the trial were considered failures from time of drop-out onwards, irrespective of whether their smear converted at the time they dropped out.

Values for sensitivity, specificity, positive predictive value (PPV), and negative predictive value (NPV) are provided in Table 3. The data show that S_24_ is fairly specific for T with values of 100% for BDQ and 86. 2% for control, respectively, while sensitivity is much lower, i.e., 56% for BDQ and 64. 9% for control. Specificity, PPV, and NPV are consistently higher for S_24_ than for S_8_ regardless of treatment assignment.

**Table 3:** Sensitivity, specificity, positive predictive value and negative predictive value of S_8_ and S_24_.

	Sensitivity ^(1)^	Specificity^(2)^	PPV^(3)^	NPV^(4)^
**BDQ 8W (S**_**8**_**)**	56.0% = 14/25	63.4% = 26/41	48.3% = 14/29	70.3% = 26/37
**Placebo 8W (S**_**8**_**)**	75.7% = 28/37	41.4% = 12/29	62.2% = 28/45	57.2% = 12/21
**BDQ 24W (S**_**24**_**)**	56.0% = 14/25	100% = 41/41	100% = 14/14	78.8% = 41/52
**Placebo 24W (S**_**24**_**)**	64.9% = 24/37	86.2% = 25/29	85.7% = 24/28	65.8% = 25/38

(1) Sensitivity = true positive/(true positive + false negative) = proportion of patients with persistent positive cultures among those in whom treatment failed

(2) Specificity = true negative/(true negative + false positive) = proportion of patients with initial culture conversion among those with successful treatment outcome

(3) PPV = true positive/(true positive + false positive) = proportion of patients with positive cultures in whom treatment failed among all those with persistent culture positivity

(4) NPV = true negative/(true negative + false negative) = proportion of converters in whom treatment was successful among those with initial culture conversion

### Re-analysis of the primary endpoint using multiple imputation

Among the 132 patients, 5 (3.8%) patients dropped out prior to Week 8 and an additional 17 (12.9%) patients dropped out between Week 8 and Week 24. A total of 85 (64.4%) patients completed the trial. It is further noted that dropout is monotone, i.e., response rates are available for all patients at all time points until the time of drop out. An overview of the number of observed (O) and missing (M) patients is presented in Table 4.

**Table 4:** Drop-out rate during the trial.

	Week						
Drop out, n/N (%)	8	24	36	48	60	72	120
85/132 (64.4%)	O	O	O	O	O	O	O
11/132 (8.3%)	O	O	O	O	O	O	M
6/132 (4.5%)	O	O	O	O	O	M	M
5/132 (3.8%)	O	O	O	O	M	M	M
3/132 (2.3%)	O	O	O	M	M	M	M
0/132	O	O	M	M	M	M	M
17/132 (12.9%)	O	M	M	M	M	M	M
5/132 (3.9%)	M	M	M	M	M	M	M

Table 5 shows that the vast majority of patients at later time points were responders. Indeed, in the most extreme case, the response rate (M = F) in the BDQ group at Week 120 was 62% while another 35% of patients were failures as a result of drop out. This implies that for patients who were observed to complete the trial only 3% did not reach culture conversion, while for 35% of patients their outcome was not observed at trial end.

**Table 5:** Response rates (M = F) and % missing data during the trial.

		Week						
		8	24	36	48	60	72	120
**Placebo (N = 66)**	Response (%)/missing (%)	32/6	56/20	61/20	64/21	58/26	56/30	44/36
**BDQ(N = 66)**	Response (%)/missing (%)	56/2	79/14	73/14	74/17	73/20	71/24	62/35

As the primary endpoint used a missing = failure approach as a single imputation which artificially enhances the association in the evaluation of surrogacy for early dropouts, a multiple imputation analysis was performed as a sensitivity analysis. Given that the missing data pattern is monotone and consists of binary outcome variables (response/ no response) [23], a logistic regression model was fitted for each time point, with the previous time points and treatment group as covariates. This way, the response variable at Week 8 was only regressed for treatment. The response variable at Week 24 was fitted using Week 8 response and treatment as independent variables etc… The imputed observations were subsequently obtained using the imputation algorithm as described in van Buuren, S 2012 [23]. A total of 5 imputations were deemed sufficient.

**Fig 2:**
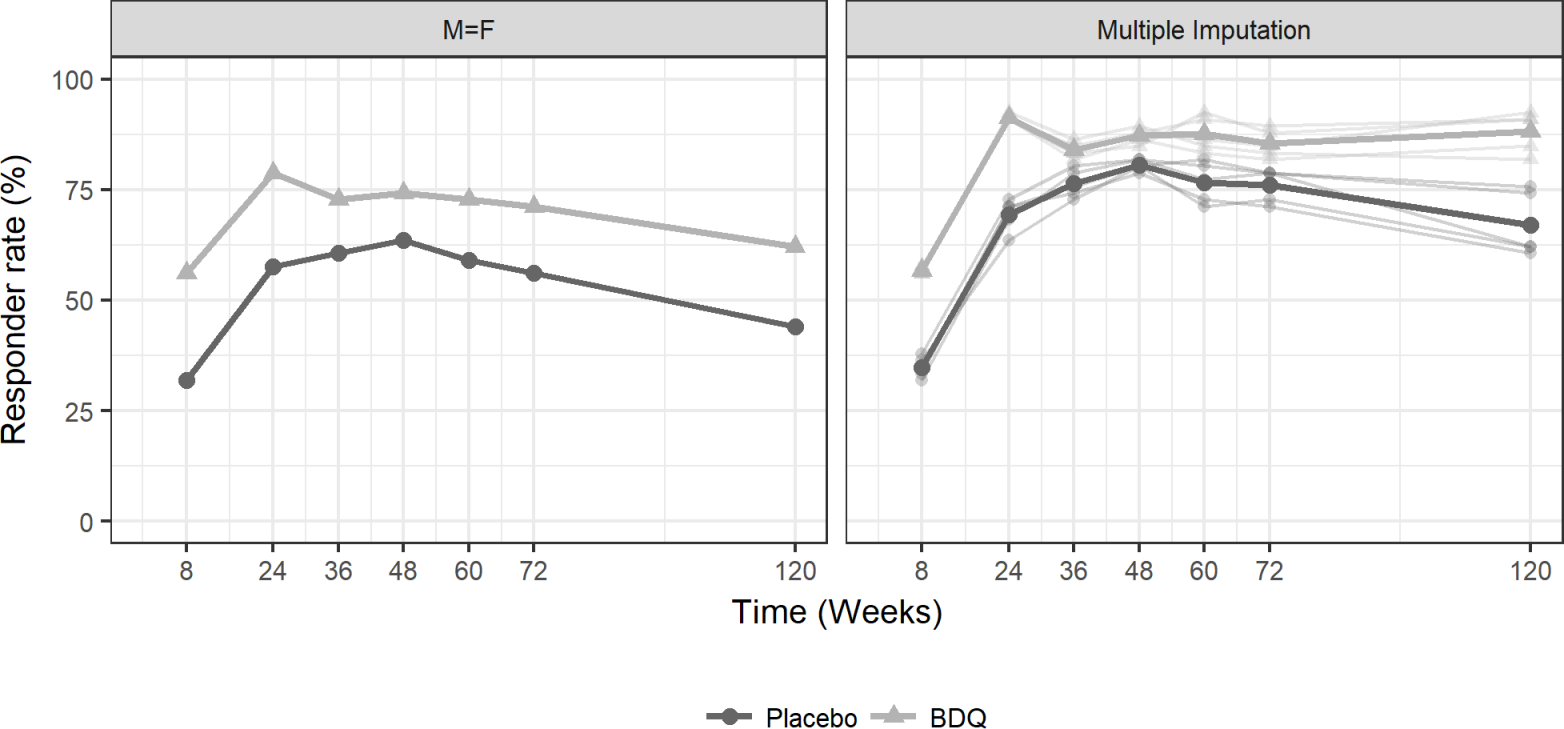
Response rate over time in the missing = failure and multiple imputation analyses.

The results indicate that application of the multiple imputation increased the response rates at all time points as observed in Fig 2. In addition, the imputed profiles behaved consistently. The average of the 5 imputations at Week 8, Week 24 and Week 120 are displayed in Table 6.

**Table 6:** Average response rate over time in the multiple imputation analysis.

		Week						
		8	24	36	48	60	72	120
**Placebo** **(N = 66)**	Response (%)	34.9	69.4	76.4	80.6	76.7	76.0	67.0
**BDQ** **(N = 66)**	Response (%)	56.7	91.2	83.9	87.3	87.6	85.5	88.2

S3 Table presents the relationship between S_24_ and T for BDQ and between S_24_ and T for placebo, based on culture conversion using the multiple imputation method.

In the next section, individual-level surrogacy using the Week 8 and Week 24 interim results as putative surrogate endpoints is evaluated. The multiple imputations serve as a sensitivity analysis to gain additional insights.

### Individual causal association

**Fig 3:**
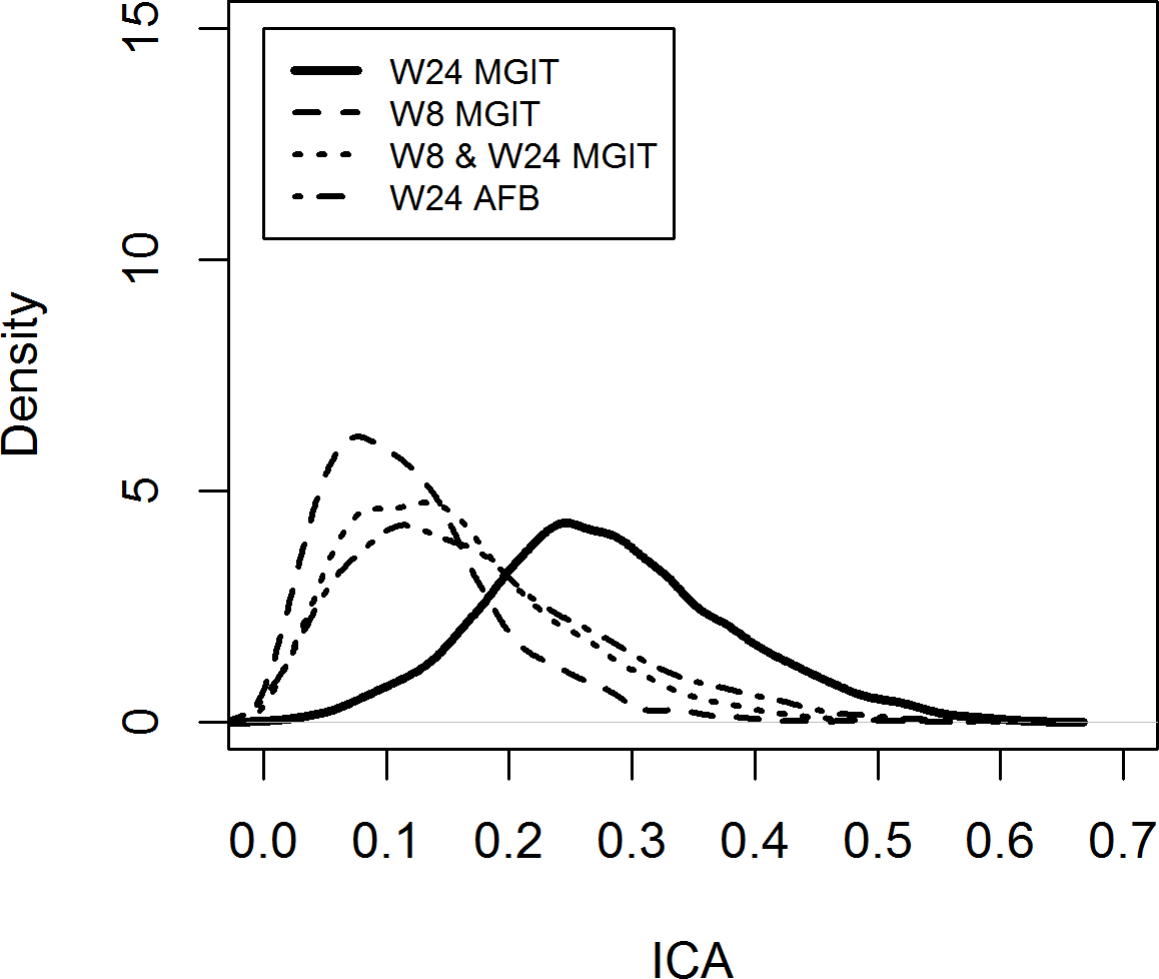
Densities and distribution of the individual causal association between ΔT and ΔS for S_8_, S_8_xS_24_, S_24_ based on MGIT, and S_24_ based on AFB.

The ICA for each of the two putative surrogate endpoints S_8_ and S_24_ can be assessed using the R library *Surrogate*, which is available in CRAN [26]. The R code used is available upon request. In addition to the two binary surrogate endpoints S_8_ and S_24_, we also investigated a third putative surrogate endpoint, denoted as S_8_×S_24_, where success is defined as culture conversion at *both* Week 8 and Week 24, and a fourth putative surrogate endpoint, which is based on AFB, rather than MGIT. The densities of the ICA with distributional statistics for each of the four binary surrogate endpoints are depicted in Fig 3.

S_24_ performs consistently better than S_8_ and S_8_×S_24_, both of which perform similarly (Table 7). It is clear from above analysis that S_24_ is the most predictive surrogate even though the density of ICA is also relatively low for S_24_. In addition, culture conversion at Week 24 was more predictive than smear conversion.

**Table 7:** Distribution of individual causal association between ΔT and ΔS for S_8_, S_8_xS_24_, S_24_ based on MGIT, and S_24_ based on AFB.

	Percentiles of the distribution						
	5%	10%	20%	50%	80%	90%	95%
**ICA (S**_**8**_**)**	0.028	0.041	0.059	0.108	0.171	0.216	0.256
**ICA** **(S**_**8**_**×S**_**24**_**)**	0.040	0.056	0.079	0.143	0.226	0.278	0.321
**ICA (S**_**24**_**)**	0.126	0.161	0.199	0.273	0.364	0.417	0.460
**ICA**_**AFB**_ **(S**_**24**_**)**	0.041	0.057	0.086	0.157	0.258	0.320	0.373

**Fig 4:**
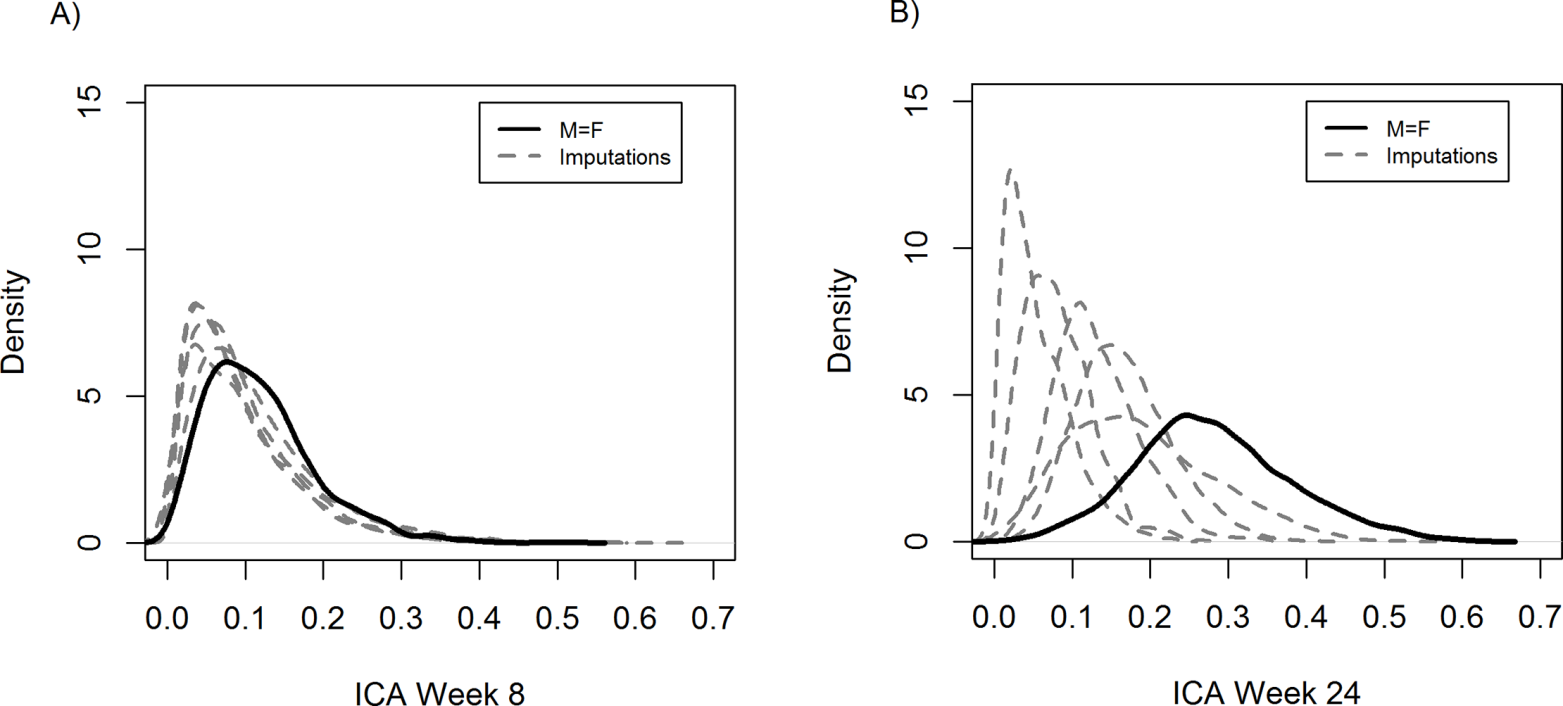
**ICA evaluated using the missing = failure and multiple imputation methods at A) Week 8 and B) Week 24**.

Sensitivity analyses evaluating the ICA using the multiple imputation method revealed that the result worsened for Week 24 culture conversion as the surrogate endpoint compared with the missing = failure method. The impact of missing data on the Week 24 surrogate endpoint was larger than on the Week 8 surrogate endpoint (Fig 4). In addition, large differences were observed among the ICA densities of the imputed data sets. Note however that there were also large differences in odds ratios as shown in the S3 Table.

### Surrogate predictive function

**Fig 5:**
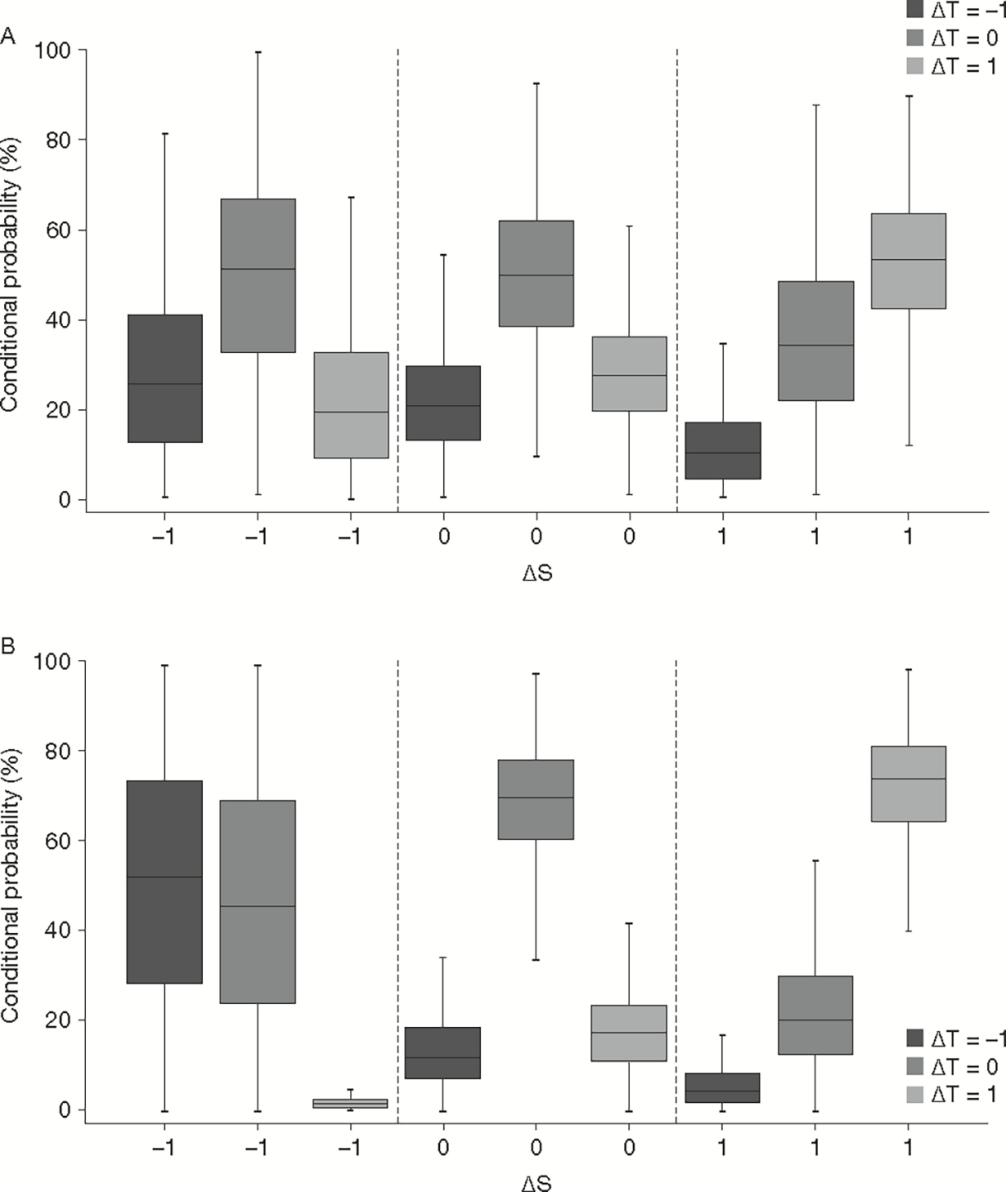
**Surrogate predictive value for A) S**_**8**_ **and B) S**_**24**_. The conditional probabilities of (A) ΔT given ΔS_8_ or (B) ΔT given ΔS_24_ are shown. For any individual patient, ΔT and ΔS will be -1 (Harm), 0 (Equal), and 1 (Benefit).

Fig 5 shows conditional probabilities of possible outcomes for the treatment effect on the true endpoint (ΔT), given the effect on the surrogate endpoint (ΔS_8_ or ΔS_24_). This graphical representation of the SPF provides more granular insight as to how predictive ΔS_24_ is for ΔT. From the nine conditional probabilities of ΔT given ΔS_24_ depicted in Fig 5, it is apparent that two conditional probabilities, i.e., P(ΔT = 1|ΔS_24_ = -1) and P(ΔT = -1|ΔS_24_ = 1) are very low, which has a straightforward interpretation. Indeed, a harmful effect of BDQ on the surrogate endpoint rules out a beneficial effect of BDQ on the true endpoint. The converse is also true, i.e., a beneficial effect of BDQ on the surrogate endpoint is unlikely to result in a harmful effect of BDQ on the true endpoint. These probabilities remained low after multiple imputation of the missing data.

Conversely, the two probabilities that were clearly highest were P(ΔT = 1|ΔS_24_ = 1) and P(ΔT = 0|ΔS_24_ = 0). This means that patients in whom BDQ will be beneficial at the surrogate endpoint (i.e, would have a positive response with BDQ but not with placebo) are also expected to benefit from BDQ at the true endpoint (ΔT = 1|ΔS_24_ = 1), with a median probability of 74%. Patients in whom the outcome on BDQ and placebo was equal at the surrogate endpoint, (i.e., would fail on either treatment or alternatively respond to either treatment) are expected to also have an equal outcome at the true endpoint (ΔT = 0|ΔS_24_ = 0), with a median probability of 70%. Note that the latter median probabilities P(ΔT = 0|ΔS_24_ = 0) were further increased to 81%, 73%, 76%, 79% and 78% respectively using the imputed data. In contrast, the median probabilities of P(ΔT = 1|ΔS_24_ = 1) substantially decreased to 37%, 66%, 62%, 21% and 58% respectively, at the expense of comparable increases of the median probabilities of P(ΔT = 0|ΔS_24_ = 1).

A less clear picture was seen from ΔS_8_, as all median probabilities were in the range of 10% to 50%, which makes it difficult to draw firm conclusions as to which value of ΔS_8_ is likely (or unlikely) to correspond with a value of ΔT.

## Discussion

In the BDQ registrational Phase II trials (C208 Stage II and C209) [19,27], the selection of Week 24 instead of Week 8 culture conversion as an interim endpoint was based on a number of considerations. First, the treatment duration of BDQ or placebo (added to a preferred five-drug MDR-TB regimen) was 24 weeks, and it was plausible to evaluate at the end of the Week 24 treatment period. We also anticipated that culture conversion in patients with MDR-TB would generally be slower than in patients with DS-TB [28]. Another argument in favor of a Week 24 interim endpoint was our belief that culture in liquid media would be more sensitive than traditionally used solid media and paradoxically increase sputum culture conversion times. In light of the results presented here, we conclude that the selection of 24 weeks as an interim endpoint was a reasonable approach.

The current work sought to compare Week 8 versus Week 24 sputum culture conversion as a surrogate endpoint for favorable outcome at Week 120 in the treatment of MDR-TB that was based on the results of a single Phase II trial. Our results show that Week 8 culture conversion is a poor surrogate whereas Week 24 culture conversion performs better, in terms of specificity, PPV, NPV, and the ICA and SPF. This finding is important both for the design of future MDR-TB trials and in the context of individual patient care where failure to culture convert may prompt re-evaluation of ‘a failing regimen’. Even though the ICA was relatively low for the Week 24 time point, the analysis based on the SPF clearly shows that some situations can be confidently ruled out. For instance, it seems to be very unlikely that a beneficial effect of BDQ on Week 120 culture conversion (the true endpoint) is to be expected given a harmful effect at Week 24 (the surrogate endpoint). Conversely, a beneficial effect of BDQ on Week 24 culture conversion (surrogate endpoint) is unlikely to result in a harmful effect at Week 120 (true endpoint).

The relatively low ICA values are not surprising for a number of reasons. First, substantial information is lost in dichotomizing surrogate endpoints (i.e., S_8_, and S_24_). Alternative surrogate endpoints such as ‘time to sputum culture conversion’ and rate of bacterial load decline may perform better but require further investigation [19,29]. A methodology to evaluate continuous surrogate endpoints in combination with binary true endpoints is currently being developed and will allow evaluation of these putative surrogates with S_24_, using the ICA as a common measure of association. Another reason for the low ICA density is the relatively high number of patients on placebo who either converted after Week 24 or who relapsed towards the end of the trial. Finally, a sensitivity analysis using multiple imputation has demonstrated that the high rate of dropout may artificially enhance the agreement between S_24_ and the true endpoint (Week 120), resulting in elevated values for the ICA.

In retrospect, given that C208 Stage II had an add-on superiority design during which BDQ was added for 24 weeks to a standardized background regimen, acceptance of culture conversion at 24 weeks as a surrogate endpoint appears to have been a reasonable approach. BDQ not only shortened the time to culture conversion but also prevented relapse many months after treatment with BDQ stopped, in a setting where both treatment groups had a background regimen that was similar in composition and duration, acknowledging that more changes to the background regimen were made in the placebo group [19].

The field of MDR-TB treatment is evolving. In 2016, the WHO recommended the 9-month short-course regimen for the treatment of uncomplicated MDR-TB [30]. Various clinical trial initiatives are looking at shortened, simplified regimens for treatment of MDR-TB, including the endTB initiative (NCT02754765), TB PRACTECAL (NCT02589782), and Nix-TB (NCT02333799) [31], looking at 6–9 month, all oral regimens containing three to five drugs, most of which combine potent new drugs with existing or repurposed group five drugs [28]. As the field moves away from the current 18–24 month regimens and towards newer simplified short-course regimens composed of several strong drugs, it is possible that time to culture conversion will occur earlier, possibly nearing 100% by 2 months akin to DS-TB, in which case a 6-month surrogate endpoint may no longer have an advantage over the 2-month surrogate endpoint [28,31,32].

## Supporting information

S1 Table Institutional review boards. (DOCX)

S2 Table A) Relationship between S_24_ (on the basis of AFB smear conversion) and T for BDQ; B) Relationship between S_24_ (on the basis of AFB smear conversion) and T for Placebo control.
(DOCX)

S3 Table A) Relationship between S_24_ (on the basis of culture conversion) and T for BDQ: imputed values; B) Relationship between S_24_ (on the basis of culture conversion) and T for Placebo control: imputed values.
(DOCX)
