# Supplementary material for: Evaluation of six months sputum culture conversion as a surrogate endpoint in a multidrug resistant-tuberculosis trial
Source: PLoS One. 2018 Jul 19;13(7):e0200539. doi: 10.1371/journal.pone.0200539 (PMC6053142; doi:10.1371/journal.pone.0200539)
Supplement: S1 Table — (DOCX) [file pone.0200539.s001.docx]

| **Table 1. Institutional Review Boards** |
| --- |
| *Pharma Ethics (Pty) Ltd.*  *123 Amcor Road*  *Lyttelton Manor*  *Pretoria*  *0157*  *South Africa* |
| *The university of KwaZulu-Natal Biomedical Research Ethics Administration research Office*  *RooomN40*  *Govan Mbeki Building*  *University Road*  *Westville Campus*  *KwaZulu-Natal*  *South Africa* |
| *Research Ethics Committee*  *University of Cape Town*  *Faculty of Health Sciences*  *E52-23 Old Main Building*  *Groote Schuur Hospital*  *Observatory*  *7925*  *South Africa* |
| *BioMedical Research Ethics Administration*  *Nelson R Mandela School of Medicine*  *University of Kwazulu-Natal*  *Westville Campus*  *Govan Mbeki Building*  *Private Bag X54001*  *Durban*  *4000*  *Kwazulu- Natal*  *South Africa* |
| *Wits Ethics Secretariat Office*  *C/O Wits Health Consortium (Pty) Ltd*  *8 Blackwood Avenue*  *Parktown*  *Johannesburg*  *2193*  *South Africa* |
| *COMITÉ DE ETICA PARA LA INVESTIGACIÓN DE LA FACULTAD DE MEDICINA DE LA UNIV. DE SAN MARTIN DE PORRES*  *Av Alameda Del Corregidor 1531 Urb Los Sirius Las, La Molina*  *Lima 12*  *Peru* |
| *Comite Institucional de Etica de la Universidad Peruana Cayetano Heredia*  *Av. Honorio Delgado 430*  *Lima 31*  *Peru* |
| *Comite de Etica en Investigacion Biomedica del Hospital Nacional Dos de Mayo*  *Parque Historia de la Medicina Peruana s/n. Alt. Cdra.13*  *Av. Grau*  *Cercado de Lima*  *Peru* |
| *Comite Institucional de Etica en Investigacion del Hospital Maria Auxiliadora*  *Av. Miguel Iglesias 968, San Juan de Miraflores*  *Lima 29*  *Peru* |
| *Ethics Committee*  *Smt. NHL Municipal Medical College*  *Ellisbridge*  *Ahmedabad – 380006*  *India* |
| *Ethics Committee*  *National institute for Research in Tuberculosis*  *(Indian Council of Medical Research),*  *No. 1, Sathyamoorthy Road, Chetput*  *Chennai-600031, Tamil Nadu*  *India* |
| *Ethics Committee*  *All India Institute of Medical Sciences*  *Ansari Nagar, New Delhi – 110029*  *India*  *Rajan Babu Institute for Pulmonary Medicine & Tuberculosis (RBIPMT), Kingsway Camp, G.T.B. Nagar, Delhi 110009, India* |
| *Independent Interdisciplinary Committee on Ethical Expertise of Clinical Studies*  *Leningradsky prospect, 51 Moscow 125468*  *Russia* |
| *The Ethics Committee for Clinical Trial son Medicinal Products*  *Aizkraukles Street 21-113*  *Riga, LV-1006*  *Latvia* |
| *Ethics Committee of Shanghai Pulmonary Hospital*  Zhemgmin Rd. No 507  200433 Shanghai  China |
| *Independent Ethic Committee of Beijing Chest Hospital*  *No 97 Machang Tongzhou*  *101149 Beijing*  *China* |
| *Independ Ethics Committee of Shangdong Provincial Chest Hospital*  *Lishan Rd. No 46*  *250013 Jinan*  *China* |
| *Research Ethics Committee*  *Faculty of Medicine*  *Chiang Mai University*  *110 Intravaroros Road*  *Muang, Chiang Mai 50200*  *Thailand* |
| *Ethics Committee*  *LEC:*  *The Institutional Review Board*  *Maharat Nakhon Ratchasima H ospital*  *49 Medical Education Center*  *Changpuek Rd. Tambol Naimuang*  *Amphur Muang, Nakhonratchasima 30000*  *Thailand*  *CEC:*  *The Ethical Review Committee for Research in Human Subjects*  *Ministry of Public Health*  *Tiwanon Road, Muang, Nonthaburi 11000*  *Thailand* |
| *Ethics Committee*  *LEC:*  *Ethical Review Committee of Chest Disease Institute*  *9^th^ Building, 5^th^ Floor, 39 Tiwanon Road*  *Tambol Bangkrasao*  *Amphur Muang, Nonthaburi 11000*  *Thailand*  *CEC:*  *The Ethical Review Committee for Research in Human Subjects*  *Ministry of Public Health*  *Tiwanon Road, Muang, Nonthaburi 11000*  *Thailand* |
| *QUEZON INSTITUTE CLUSTER ETHICS REVIEW COMMITTEE*  *Quezon Avenue*  *Quezon City, 1100*  *Philippines* |
| *Ethics Review Committee*  *Department of Research and Development*  *Lung Center of the Philippines*  *Quezon Avenue, Quezon City 1100*  *Philippines* |
| *RESEARCH INSTITUTE FOR TROPICAL MEDICINE INSTITUTIONAL REVIEW BOARD*  *Alabang*  *Muntinlupa City 1781*  *Philippines* |
| *KEMRI Ethical Review Committee*  *Kenya Medical Research Institute*  *KEMRI Headquarters*  *Mbagathi Way*  *Nairobi, 00200*  *Kenya* |
| *LEC:*  *Comite de Etica em Pesquisa do Instituto de Pesquisa*  *Clinica Evandro Chagas IPEC-FIOCRUZ*  *Av. Brasil, 4365*  *Manguinhos – Rio de Janeiro – RJ – 21040-900*  *Brazil*  *National EC:*  *Ministerio da Saude – Conselho Nacional de Saude*  *CONEP- Comissao Nacional de Etica em Pesquisa*  *Esplanada dos Ministerios, Bloco “G” – Ed. Anexo, Ala “B” – lo andar – sl. 145*  *Brasilia – DF – 70058-900*  *Brazil* |
