# Supplementary material for: Evaluation of six months sputum culture conversion as a surrogate endpoint in a multidrug resistant-tuberculosis trial
Source: PLoS One. 2018 Jul 19;13(7):e0200539. doi: 10.1371/journal.pone.0200539 (PMC6053142; doi:10.1371/journal.pone.0200539)
Supplement: S2 Table — A) Relationship between S24 (on the basis of AFB smear conversion) and T for BDQ; B) Relationship between S24 (on the basis of AFB smear conversion) and T for Placebo control. (DOCX) [file pone.0200539.s002.docx]

**Table 2A. Relationship between S_24_ (on the basis of AFB smear conversion) and T for BDQ**

| **Control** | | **Surrogate endpoint (S_24_)** | |
| --- | --- | --- | --- |
|  |  | **No culture conversion** | **Culture conversion** |
| **True**  **endpoint (T)** | **No culture conversion** | 9 | 16 |
|  | **Culture conversion** | 5 | 36 |
|  | | | |

**Table 2B. Relationship between S_24_ (on the basis of AFB smear conversion) and T for Placebo control**

| **BDQ** | | **Surrogate endpoint (S_24_)** | |
| --- | --- | --- | --- |
|  |  | **No culture conversion** | **Culture conversion** |
| **True**  **endpoint (T)** | **No culture conversion** | 22 | 15 |
|  | **Culture conversion** | 3 | 26 |
