# Supplementary material for: Evaluation of six months sputum culture conversion as a surrogate endpoint in a multidrug resistant-tuberculosis trial
Source: PLoS One. 2018 Jul 19;13(7):e0200539. doi: 10.1371/journal.pone.0200539 (PMC6053142; doi:10.1371/journal.pone.0200539)
Supplement: S3 Table — A) Relationship between S24 (on the basis of culture conversion) and T for BDQ: imputed values; B) Relationship between S24 (on the basis of culture conversion) and T for Placebo control: imputed values. (DOCX) [file pone.0200539.s003.docx]

| **Table 3A. Relationship between S_24_ (on the basis of culture conversion) and T for BDQ: imputed values**   \| **Control** \| \| **Surrogate endpoint (S_24_)** \| \| \| --- \| --- \| --- \| --- \| \|  \|  \| **No culture conversion** \| **Culture conversion** \| \| **True**  **endpoint (T)** \| **No culture conversion** \| 8,13,14,6,16 \| 9,13,11,10,9 \| \| **Culture conversion** \| 10,6,7,13,8 \| 39,34,34,37,33 \| \|  \| \| \| \|   **Table 3B. Relationship between S_24_ (on the basis of culture conversion) and T for Placebo control: imputed values** |
| --- | --- | --- | --- | --- | --- | --- | --- | --- | --- | --- | --- | --- | --- | --- | --- | --- | --- | --- | --- |

| **BDQ** | | **Surrogate endpoint (S_24_)** | |
| --- | --- | --- | --- |
|  |  | **No culture conversion** | **Culture conversion** |
| **True**  **endpoint (T)** | **No culture conversion** | 2,1,4,1,2 | 4,4,6,5,10 |
|  | **Culture conversion** | 4,5,2,5,3 | 56,56,54,55,51 |
|  | | | |
